# Ancestral origin of *ApoE ε4* Alzheimer disease risk in Puerto Rican and African American populations

**DOI:** 10.1371/journal.pgen.1007791

**Published:** 2018-12-05

**Authors:** Farid Rajabli, Briseida E. Feliciano, Katrina Celis, Kara L. Hamilton-Nelson, Patrice L. Whitehead, Larry D. Adams, Parker L. Bussies, Clara P. Manrique, Alejandra Rodriguez, Vanessa Rodriguez, Takiyah Starks, Grace E. Byfield, Carolina B. Sierra Lopez, Jacob L. McCauley, Heriberto Acosta, Angel Chinea, Brian W. Kunkle, Christiane Reitz, Lindsay A. Farrer, Gerard D. Schellenberg, Badri N. Vardarajan, Jeffery M. Vance, Michael L. Cuccaro, Eden R. Martin, Jonathan L. Haines, Goldie S. Byrd, Gary W. Beecham, Margaret A. Pericak-Vance

**Affiliations:** 1 John P. Hussman Institute for Human Genomics, Miller School of Medicine, University of Miami, Miami, Florida, United States of America; 2 Universidad Central del Caribe, Bayamón, Puerto Rico, United States of America; 3 Center for Outreach in Alzheimer’s, Aging and Community Health at North Carolina A&T State University, Greensboro, North Carolina, United States of America; 4 Clinica de la Memoria, San Juan, Puerto Rico, United States of America; 5 Gertrude H. Sergievsky Center, Taub Institute for Research on the Aging Brain, Departments of Neurology, Psychiatry, and Epidemiology, College of Physicians and Surgeons, Columbia University, New York, New York, United States of America; 6 Departments of Medicine (Biomedical Genetics), Neurology, Ophthalmology, Epidemiology, and Biostatistics, Boston University Schools of Medicine and Public Health, Boston, Massachusetts, United States of America; 7 Penn Neurodegeneration Genomics Center, Department of Pathology and Laboratory Medicine, University of Pennsylvania Perelman School of Medicine, Philadelphia, Pennsylvania, United States of America; 8 Dr. John T. MacDonald Foundation Department of Human Genetics, Miller School of Medicine, University of Miami, Miami, Florida, United States of America; 9 Department of Population & Quantitative Health Sciences, Institute for Computational Biology, Case Western Reserve University School of Medicine, Cleveland, Ohio, United States of America; Stanford University School of Medicine, UNITED STATES

## Abstract

The *ApoE* ε4 allele is the most significant genetic risk factor for late-onset Alzheimer disease. The risk conferred by ε4, however, differs across populations, with populations of African ancestry showing lower ε4 risk compared to those of European or Asian ancestry. The cause of this heterogeneity in risk effect is currently unknown; it may be due to environmental or cultural factors correlated with ancestry, or it may be due to genetic variation local to the *ApoE* region that differs among populations. Exploring these hypotheses may lead to novel, population-specific therapeutics and risk predictions. To test these hypotheses, we analyzed *ApoE* genotypes and genome-wide array data in individuals from African American and Puerto Rican populations. A total of 1,766 African American and 220 Puerto Rican individuals with late-onset Alzheimer disease, and 3,730 African American and 169 Puerto Rican cognitively healthy individuals (> 65 years) participated in the study. We first assessed average ancestry across the genome (“global” ancestry) and then tested it for interaction with *ApoE* genotypes. Next, we assessed the ancestral background of *ApoE* alleles (“local” ancestry) and tested if ancestry local to *ApoE* influenced Alzheimer disease risk while controlling for global ancestry. Measures of global ancestry showed no interaction with *ApoE* risk (Puerto Rican: p-value = 0.49; African American: p-value = 0.65). Conversely, ancestry local to the *ApoE* region showed an interaction with the *ApoE* ε4 allele in both populations (Puerto Rican: p-value = 0.019; African American: p-value = 0.005). *ApoE* ε4 alleles on an African background conferred a lower risk than those with a European ancestral background, regardless of population (Puerto Rican: OR = 1.26 on African background, OR = 4.49 on European; African American: OR = 2.34 on African background, OR = 3.05 on European background). Factors contributing to the lower risk effect in the *ApoE* gene ε4 allele are likely due to ancestry-specific genetic factors near *ApoE* rather than non-genetic ethnic, cultural, and environmental factors.

## Author summary

The strongest risk gene identified for late-onset Alzheimer disease is *ApoE*. However, the risk for Alzheimer disease due to *ApoE* is not consistent across populations. For example, individuals with African ancestry experience less risk from *ApoE* ε4 than individuals of European or Asian ancestry. The cause of the difference in risk effect is currently unknown. This has led us to ask: What is/are the factor(s) contributing to the risk effect variation of *ApoE* across the populations? We hypothesized two possibilities for the variability of *ApoE* risk: 1) ethnic-related environmental factors that vary across populations, such as diet and lifestyle activities, or 2) a population-specific genetic difference in the *ApoE* gene, or in its surrounding region. We tested our hypothesis using populations with more than one genetic ancestral background, specifically African Americans and Puerto Ricans. Our study showed that the risk of Alzheimer disease is lower for individuals who inherited the genomic region surrounding the *ApoE* gene from an African ancestor than it is for risk allele carriers who inherited the region from a European ancestor. These findings suggest that protective genetic variant(s) most likely lie(s) within the genetic region surrounding the *ApoE* gene on the African ancestral background.

## Introduction

Late-onset Alzheimer disease (LOAD) is a progressive neurodegenerative disorder characterized by loss of memory and other cognitive functions. It is the most common form of dementia worldwide [1], with prevalence increasing with age (e.g., ~30–40% by 85–89 years) [2]. The etiology of AD is multifactorial with genetic, and environmental factors all influencing risk.

The most significant genetic risk factor for LOAD is the *ApoE* gene [3,4]. Three common *ApoE* alleles have been identified (ε2, ε3, and ε4). The ε3 allele is the most frequent and is typically considered “neutral” regarding AD risk. The *ApoE* ε4 allele both increases the risk and decreases the age-of-onset of developing AD [4]. Conversely, the ε2 allele is protective against AD [4,5]. Although *ApoE* is an AD risk factor in nearly all populations, the risk of AD for ε4 carriers differs among racial/ethnic groups [6]. The strongest reported risk for ε4 allele is in East-Asian populations (ε3/ε4 odds ratio OR: 3.1–5.6; ε4/ε4 OR: 11.8–33.1) [6,7] followed by non-Hispanic Whites (NHW) (ε3/ε4 odds ratio [OR]: 3.2; ε4/ε4 OR: 14.9) [6,8–10] with a considerably lower risk to develop AD for an ε4 carrier in African-Ancestry populations, such as African Americans (AA) and Caribbean Hispanics (CHI). Studies in African-ancestry cohorts consistently reported significant association between *ApoE* ε4 homozygosity and AD, but showed inconsistent results for ε4 heterozygote allele individuals (ε3/ε4 OR:1.1–2.2; ε4/ε4 OR: 2.2–5.7) [6,8–13]. The reason for this heterogeneous risk effect of *ApoE* is currently unknown. This disparity in risk may be due to ethnic-related environmental factors that vary across populations, such as diet and lifestyle activities, or the difference may be due to population-specific genetic factors. Exceptions include studies among the Wadi Ara and American Indian populations, but these studies may suffer from low power due to small sample sizes [14–16].

**Fig 1:**
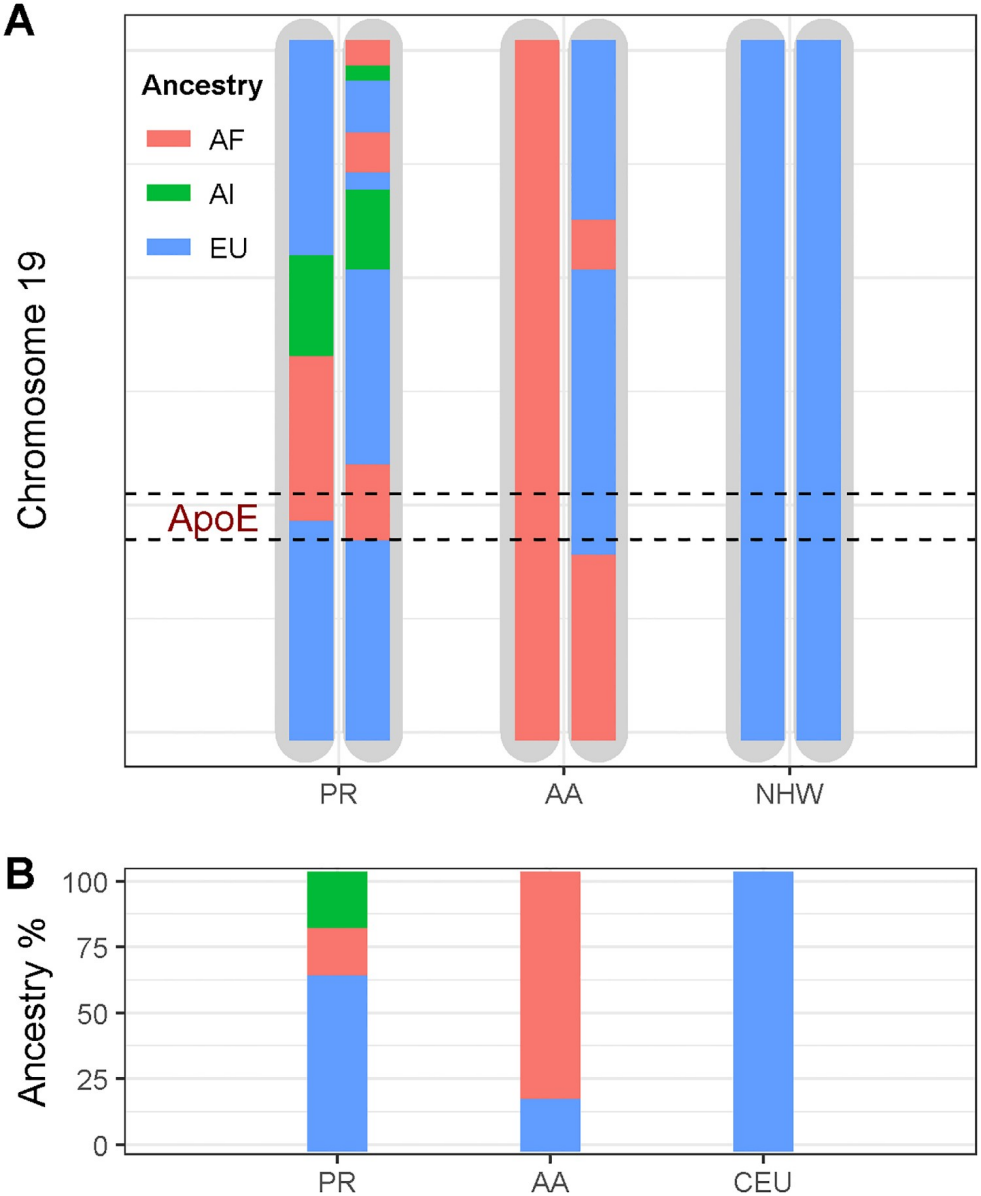
Illustration of local and global ancestry. This figure represents chromosome 19 from Puerto Rican, African American, and European ancestry individuals. (A) The colored chromosomal segments represent the admixture blocks “local” to each genomic region, with each ancestry coded by a different color (red: African (AF), blue: European (EU), green: American Indian (AI)). (B) The global ancestry estimated by the average ancestry across the whole genome. The Puerto Rican individual has one African block and one European block around *ApoE* (represented by the dashed line); that is, the local ancestry around *ApoE* is African/European for this individual. The African American individual, though mostly African genome-wide, also has African/European local ancestry at the *ApoE* gene.

Ancestral methods examining both global (GA) and local (LA) ancestry can be used to explore these different hypotheses. GA refers to an individual’s average ancestry across his/her entire genome while LA refers to the ancestral background of a particular (i.e., “local”) chromosomal region within an individual genome (Fig 1). GA is predominantly correlated with ethnic, cultural, and environmental factors that are related to broader definitions of race and ethnicity [17–20]. Conversely, LA is often correlated with ancestry-specific genetic factors that are located in or near the genomic region in question [21,22]. As such, an understanding of LA around the *ApoE* region may help inform how we interpret the race/ethnicity differences observed in ε4 risk. Specifically, if cultural and environmental effects play a major role in *ApoE* heterogeneity, we would expect GA to interact with ε4 to influence AD risk. There will also be GA and allele interaction if there is epistasis with alleles on other choromsomes that have different frequencies between ancestral populations. However, if genetic modifiers or protective factors local to the *ApoE* region (e.g., cis-acting enhancers, eQTL, etc.) play a major role in *ApoE* ε4 heterogeneity, we would expect LA to interact with ε4 to influence AD risk.

Admixed populations, due to their ancestral heterogeneity, often show complex patterns of GA and LA, enabling us to test these hypotheses. As such, we utilized two admixed populations (CHI from Puerto Rico (PR), and AA) to assess the relationship between *ApoE* ε4 risk and patterns of GA and LA. PR individuals commonly have European (EU), African (AF) and Amerindian (AI) ancestors, while AA individuals often have both EU and AF ancestors. To test the hypothesis that the population-specific risk is due to ethnic-related environmental factors that vary across populations, we compared those *ApoE* ε4 carriers who inherited most of their chromosomes from AF ancestors to those who inherited most of their chromosomes from their EU ancestors by using GA. If there are additional genomic loci outside of the *ApoE* gene contributing to the population risk difference, then individuals with the highest GA load of EU (or AF) ancestry would match the EU (or AF) population risk. Alternatively, to test the hypothesis that the disparity in risk may be due to genetic modifiers or protective factors local to *ApoE*, we compared the LAs in the admixed populations with those of the corresponding ancestral population (e.g., if one inherited his/her *ApoE* LA from the EU ancestors, his/her risk for AD would be similar to the EU population risk).

Our results strongly suggest that an ancestry-specific region surrounding the *ApoE* gene is contributing to the lower risk of AD in AA and PR ε4 carriers, supporting the hypothesis that the “protective” effect is due to the ancestry-specific genetic factors around the *ApoE* genomic region.

## Results

First, we performed two genotype-based regression tests to assess global ancestry and local ancestry interaction with *ApoE* genotype (see Methods for details). Results showed that the LA by *ApoE* interaction term (dose of AF ancestry by dose of ε4 allele; LAx*ApoE*) was significantly different from 0 in both PR and AA populations (PR: likelihood ratio test (LRT), p-value = 0.019; AA: LRT, p-value = 0.005). The effect size of the interaction term was negatively correlated with AD (PR: OR = 0.2 (CI: 0.05–0.76); AA: OR = 0.75 (CI: 0.61–0.91)). This was in contrast to the GA by *ApoE* interaction term (*GAxApoE)*, which was not significant in either PR or AA (PR: LRT, p = 0.49; AA: LRT, p-value = 0.65).

Since we identified a significant interaction, we performed a haplotype-based regression test to assess the effect size of ancestry-specific alleles (see Methods for details). We found that the effect size of the ε4 risk allele was significant across the ancestral haplotypes, even while accounting for correlations with GA (Table 1). In the PR dataset, the ε4 alleles on an EU ancestral background were significantly associated with AD (p-value = 3.7e-05; OR = 4.49) compared to ε3 alleles from an EU ancestral background. However, ε4 vs ε3 showed no significant effect on the AF LA background (p-value = 0.67; OR = 1.26). Similarly, in the AA dataset, the ε4 haplotypes of EU ancestry showed a stronger risk effect (OR = 3.05; p-value = 4.9e-17) than those in the AA dataset of AF ancestry (OR = 2.34; p-value = 9.2e-45). We tested the difference between the effect sizes of ancestral backgrounds by using t-test for means. Test results showed that effect sizes between the ancestral backgrounds are different with nominal significance in both populations (PR: p-value = 0.059; AA: p-value = 0.068). It is of note that these models all include GA as covariates, indicating that the effects seen are independent of the GA.

**Table 1:** Haplotype analysis results, *ApoE* ε3 vs *ApoE* ε4, by cohort and haplotype local ancestry.

Cohort	Haplotype Ancestry	N*	ε4 *OR*	%95 *CI*	p-value	t-test for means
Puerto Rican	European	307	4.49	2.2–9.2	3.7e-05	0.059
	African	67	1.26	0.4–3.7	6.7e-01	
African American	European	1341	3.05	2.4–3.9	4.9e-17	0.068
	African	5587	2.34	1.8–3.0	9.2e-45	

* number of haplotypes used in model

In the subgroup of individuals with homozygote ε4 and ε3 alleles, results showed that ε4 haplotypes of EU ancestry have a stronger risk effect (OR = 18.44 (CI: 9.6–35.6); p-value = 3.5e-18) than those with AF ancestry (OR = 6.48 (CI: 3.4–12.5); p-value = 4.3e-63). The t-test of means showed that effect sizes of EU and AF backgrounds are significantly different (p-value = 0.003).

**Fig 2:**
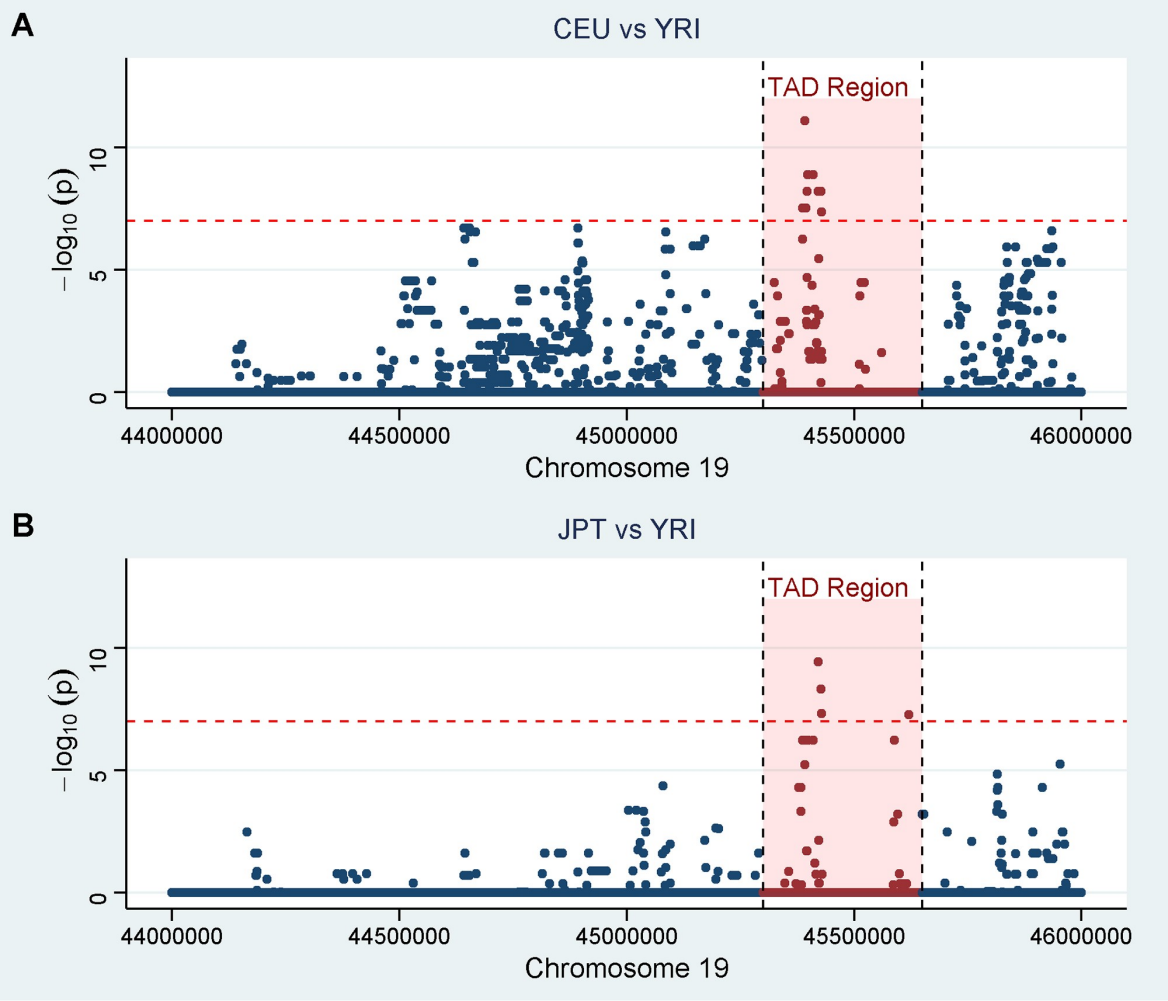
Bonferroni corrected p-values for the pairwise comparisons of the allele frequencies in 1000 Genome sequence data between (A) CEU and YRI, and (B) JPT and YRI populations. Red region represents topologically associated domain, containing *ApoE*.

Since we observed that AF ancestral background surrounding the *ApoE* gene is contributing to the lower risk of AD, we examined the genetic region surrounding *ApoE* by using 1000 Genome sequence data from three populations of the Utah Residents with Northern and Western European Ancestry (CEU), Japanese in Tokyo (JPT), and Yoruba in Ibadan (YRI). We identified 43 variants using Pearson’s chi-square test between the CEU vs. YRI and JPT vs. YRI populations, which were significant following the Bonferroni correction for multiple comparisons. Table 2 shows the list of 15 most significant variants with the Bonferroni corrected p-values less than 1 ×10^−5^. The whole list of variants is shown in the S2 Table. Fig 2 demonstrates Bonferroni corrected p-values for the pairwise comparisons between CEU and YRI, and JPT and YRI populations. The primary CEU and JPT peaks align, and lie within the strongest Topologically Associated Domain (TAD) containing the *ApoE* gene. None of the significantly different variants were in the protein-coding DNA in the defined region around the *ApoE* gene. It is noteworthy that just 6 variants in sequence data comparison showed significant difference (with the lowest p-value = 0.0052) between the CEU and JPT and all of them were found out of the TAD region containing the *ApoE*.

**Table 2:** Top list of the potential protective variants at the local ancestry blocks surrounding the *ApoE* gene.

Marker ID	Base Position	Reference Allele	Alternative Allele	Bonferroni corrected p-values	
				CEU vs. YRI	JPT vs.YRI
rs6857	45392254	C	T	8.44E-12	5.88E-06
rs157590	45398716	A	C	1.31E-09	5.85E-07
rs157588	45398264	C	T	1.31E-09	5.85E-07
rs157585	45397512	A	C	1.31E-09	5.85E-07
rs769449	45410002	G	A	1.31E-09	5.85E-07
rs157584	45396899	T	C	6.45E-09	5.85E-07
rs111789331	45427125	T	A	6.45E-09	4.75E-09
rs12721046	45421254	G	A	6.45E-09	3.71E-10
rs142042446	45386467	G	GTAA	3.02E-08	5.85E-07
rs71352238	45394336	T	C	3.02E-08	5.85E-07
rs12972156	45387459	C	G	3.02E-08	5.85E-07
rs12972970	45387596	G	A	3.02E-08	5.85E-07
rs34342646	45388130	G	A	3.02E-08	5.85E-07
rs66626994	45428234	G	A	4.37E-08	4.97E-08

## Discussion

These findings strongly support our hypothesis that genetic modifiers local to the *ApoE* region influence the risk of the ε4 allele, showing a weaker risk effect on the AF ancestral background and stronger effect on the EU ancestral background. There was no evidence that overall ancestry (GA) has an effect on the heterogeneity of *ApoE* ε4 risk within the populations, which we used as a surrogate for non-genetic cultural/ethnic differences. Additionally, we observed a stronger risk effect on the EU ε4 haplotypes (or conversely, a protective effect on AF ε4 haplotypes). This effect was especially pronounced in an analysis of ε4 homozygotes against ε3 homozygotes, a result consistent with previous reports on *ApoE* risk across populations [6,8–13].

The overlapping of the subTAD (~50kb) region and the peaks of the allele frequency differences between the CEU, JPT and YRI support the hypothesis that the variant(s) modifying ε4 risk are most likely to lie in this region. The significant differences found in non-protein-coding DNA, suggests the protective effect is due to a regulatory difference between the local ancestries. This would also suggest that possible a modifier(s) would affect *ApoE* expression itself and supports the hypothesis that the genomic region surrounding *ApoE* with AF background reduces the risk for ε4 carriers and is evidence that genetic factors may be underlying the discrepancy in ε4 allele risk effect across populations.

It should be noted that this study was not well-powered to test AI background influence on ε4 risk allele. Further research is needed to study populations with higher AI ancestral background, such as Peruvian, Mexican, and Central American populations, to understand the correlation between the AI ancestry and *ApoE*. Similarly, limitations in sample size prevented us from assessing effects in ε2 carriers.

Our findings suggest that the *ApoE* region from AF populations may contain protective factors that help mitigate the effect of the ε4 allele. In particular, comprehensive analysis of the *ApoE* region and testing for protective loci may reveal previously unappreciated biological pathways and provide translational opportunities. Research that focuses on locating protective variants represents a complementary approach to accelerating the identification of more effective targets for drug development. This, in turn, will lead to better treatments, and help reduce health disparities.

## Materials and methods

### Sources of participants

All AA cases and controls selected for genotyping were obtained from the John P. Hussman Institute for Human Genomics (HIHG) at the University of Miami Miller School of Medicine (Miami, FL), North Carolina A&T State University (Greensboro, NC), Case Western Reserve University (Cleveland, OH), and the Alzheimer’s Disease Genetic Consortium (ADGC). Samples were collected as described previously [23,24]. The AA dataset contained 1,766 AD cases (69.8% female, mean age at onset (AAO) 77.6 years [SD 8.2]) and 3,730 cognitively healthy controls (72.0% female, mean age-of-examination (AOE) 76.5 years [SD (8.3)]).

PR individuals were ascertained as a part of the Puerto Rico Alzheimer Disease and Related Disorders Initiative study. Ascertainment was focused in metropolitan areas of New York, North Carolina, Miami, and Puerto Rico. Participants were recruited and enrolled after they (or a proxy) provided written informed consent and with approval by the relevant institutional review boards. For the PR cohort, 220 unrelated cases (69.6% female, mean AAO 75.1 years [SD 9.7]) and 169 unrelated cognitively intact controls (66.4% female, mean AOE 73.6 years [SD 7.1]) were ascertained.

For both AA and PR datasets, cases were defined as individuals with AD with AAO>65 years of age; controls were defined as individuals with no evidence of cognitive problems and AOE>65 years of age. All participants were evaluated to determine case or control status based on the National Institute of Neurological and Communicative Disorders and Stroke—Alzheimer’s Disease and Related Disorders Association, criteria [25,26]. Individuals with known or suspected dementia were evaluated using the LOAD study reference [27]. Individuals who were deemed to be cognitively normal were screened with the Mini-Mental State Examination [28] or the Modified Mini-Mental State [29]. The participants were classified as AA and PR based on self-report, and the GWAS analysis confirmed these data.

### Genotyping and quality control procedures

Genome-wide single-nucleotide polymorphism (SNP) genotyping was processed on three different platforms: Expanded Multi-Ethnic Genotyping Array, Illumina 1Mduo (v3) and the Global Screening Array (Illumina, San Diego, CA, USA). *ApoE* genotyping was performed as in Saunders et al. [30]. Quality control analyses were performed using the PLINK software, v.2. [31]. The samples with a call rate less than 90% and with excess or insufficient heterozygosity (+/- 3 standard deviations) were excluded. Sex concordance was checked using X chromosome data. To eliminate duplicate and related samples, relatedness among the samples was estimated by using identity by descent (IBD). SNPs with minor allele frequencies less than 0.01 and SNPs available in samples with the call rate less than 97%, or those not in Hardy-Weinberg equilibrium (p<1.e-5), were eliminated from further analysis [32]. Further details of the QC analysis can be found in the Supplement (S1 Table).

To explore the reasons for the differences in ε4 allele risk between the populations we first assessed the genetic ancestry (LA and GA), and then tested the effect of LA and GA on the ε4 allele by building three logistic regression models.

### Assessment of genetic ancestry

To assess the LA, we phased our datasets independently applying the SHAPEIT tool ver. 2 [33] using 1000 Genomes Phase 3 reference panel [34] with default settings. We defined a region around the *ApoE* that was broad enough (chr19: 44,000,000–46,000,000) to include potential enhancers, topological associated domains, etc. while narrow enough to ensure contiguous LA blocks for most individuals in the study. After selecting the *ApoE* region, we used RFMix [35], discriminative modeling approach, to infer LA at loci across the genome. We ran RFMix with the TrioPhased option and a minimum node size of 5. We used Human Genome Diversity Project (HGDP) data as the reference panel; two for AA (EU, and AF), and three for PR (EU, AF, and AI). Then, we eliminated samples with ancestral break points across the 2Mb window (N = 892) and labeled each admixture block using the RFMix estimates. As a result, we obtained haplotype data with three LA states (AF, EU, AI) in PRs and two (AF, EU) in AAs. Afterwards, we defined haplotypes according to LA states and *ApoE* variants. S1 Fig illustrates the defining of LA at the *ApoE* gene and S2 Table shows the number of e3 and e4 alleles along AF and EU local ancestry in each population for cases and controls.

Next, we assessed GA by performing principal components analysis (PCA) using the Eigenstrat program [36]. The AA and PR datasets were combined with reference panels (using HGDP reference panels) representing diverse ancestries: EU and AF for AA, and EU, AF and AI for PR.

### Statistical analyses

To assess the effects of GA and LA on ε4 risk we used three logistic regression-based models. The first model utilized a genotype-based test to assess GA interaction with *ApoE* genotype. This model evaluated the role of GA and factors strongly correlated with GA (e.g., ethnic-related environmental factors) on *ApoE* risk variation among populations. The second model utilized a genotype-based approach to assess LA interaction with *ApoE* genotype. In this model, we examined the role of genetic modifiers or protective factors local to *ApoE* in risk variation. The third model utilized a haplotype-based approach to assess the effect sizes of ancestry-specific alleles (e.g., ε4 and ε3 alleles on the AF background) while accounting for correlations with GA. Statistical analyses were performed using the “GLM2” [37] and “GEE” [38] packages available in R computing environment.

#### Global ancestry by *ApoE* interaction

We tested the significance of the GA by *ApoE* genotype interaction (GAx*ApoE*) using the LRT. To assess the influence of the GAx*ApoE* on AD we used an age- and sex-adjusted logistic regression model. A “full model” was built that included homozygote (ε4/ε4) and heterozygote (ε3/ε4) genotypes (with ε3/ε3 being the referent) as well as measures of GA (PC1, PC2, and PC3), and GAx*ApoE* (Eq 1). This full model was tested (by the LRT) against a reduced model without the interaction terms.

AD~Age+Sex+GA+ApoE+GAxApoE (1)

#### Local ancestry by *ApoE* interaction

LA interaction was tested in a similar fashion; individuals were assigned LA “types” (for AA individuals: AF/AF, AF/EU, EU/EU; for PR individuals AF/AF, AF/EU, AF/AI, EU/EU, EU/AI, AI/AI). LA by *ApoE* interaction was tested by comparing a full model to a reduced model. The full model (Eq 2) included homozygote (ε4/ε4) and heterozygote (ε3/ε4) genotypes (with ε3/ε3 being the referent), measures of GA, LA, and LAx*ApoE* interaction term. The reduced model lacked the LAx*ApoE* interaction term.

AD~Age+Sex+LA+GA+ApoE+LAxApoE (2)

These datasets had few ε2/* genotypes and AI/* ancestral backgrounds, so individuals with these genotypes and ancestral backgrounds were excluded from the comparisons.

#### Assessment of effect sizes: Haplotype approach

A haplotype model was also tested to assess ε4 risk in an allele-specific manner. This approach tests ε4 of a particular LA background against ε3 alleles of the same background, rather than genotypes tested in the context of a LA “dose” across both parental haplotypes. To perform the analysis, ε3 and ε4 alleles were grouped by their LA (AF in one and EU in the other; the sample size of AI was too small to test adequately) and tested for association. AA and PR datasets were analyzed separately. Within each group, the effect of the ε4 allele was assessed via logistic regression using the generalized estimating equation (GEE), with principal components 1, 2, and 3 used as covariates and the individual as the grouping variable. We chose the GEE to account for the individual haplotypes correlation (since each allele is counted individually). This effectively tests the association of AF (or EU) ε4 alleles against ε3 while controlling for the effects of global ancestry, and allows us to estimate effect sizes of ancestry-specific haplotypes. In addition, we tested a haplotype-based approach among the individuals with homozygote ε4 and ε3 alleles to assess the effect size of ancestry-specific alleles on those with ε4/ε4 genotype (it was not applicable to the PR dataset since only 12 samples had homozygote ε4 alleles.). Finally, we tested the significance of difference between the effect sizes of ancestral backgrounds using t-test for means.

### Defining potential protective variants at the LA blocks around the *ApoE*

To define the potential genetic factors modifying the *ApoE* effect size we assessed the sequence differences between the ancestral backgrounds among the ε4 haplotypes. First, using the 1000 genomes database, we obtained genomic DNA sequence data from three populations of the CEU, JPT, and YRI. Secondly, we extracted the ε4 haplotypes across the defined LA block of 2 mB. In addition to EU, we tested Japanese haplotypes because ε4 allele in East Asian populations has a high-risk effect as well [6,7]. Then, we performed Pearson’s chi-square test using allele frequencies at the region of interest among the populations (CEU vs. YRI and JPT vs. YRI) to identify the list of significantly different variants that likely contain the protective variant(s). We assessed the allele frequency difference on ε3 and ε4 haplotypes separately. To make a list of ε4 haplotype-specific alleles with the significantly different frequencies we removed those that showed significant difference also among the ε3 haplotypes. Finally, we performed the Bonferroni correction [39] for the multiple comparisons.

## Supporting information

S1 Table Number of individuals and SNPs excluded after QC analysis. (DOCX)

S2 Table Counts of ε3 and ε4 alleles along African and European local ancestries in African Americans and Puerto Ricans for cases and controls. (DOCX)

S3 Table The complete list of the potential protective variants at the local ancestry blocks surrounding the *ApoE* gene. (DOCX)

S1 Fig This figure illustrates how local ancestry is estimated at *ApoE*. (A) Phasing, selecting the *ApoE* region, and classifying the haplotypes into three groups: ε2, ε3, ε4 haplotypes. (B) Building reference panels and inferring the local ancestry by using RFMix. Haplotypes classified as Reference ancestries ε2, ε3, ε4 haplotypes and admixed ε2, ε3, ε4 haplotypes. (C) Building statistical model to test the association of ancestry-aware ε4 alleles against ε3 within AF and EU subgroups, and admixed haplotypes within *ApoE* were excluded from analysis.
(TIF)
