# Supplementary material for: Ancestral origin of ApoE ε4 Alzheimer disease risk in Puerto Rican and African American populations
Source: PLoS Genet. 2018 Dec 5;14(12):e1007791. doi: 10.1371/journal.pgen.1007791 (PMC6281216; doi:10.1371/journal.pgen.1007791)
Supplement: S1 Table — (DOCX) [file pgen.1007791.s001.docx]

**S1 Table.** Number of individuals and SNPs excluded after QC Analysis

|  | **Excluded** | |
| --- | --- | --- |
| **SAMPLE QC** | **AA** | **PR** |
| Call rate < 90% | 3 | 1 |
| Heterozygosity Rate +/-3SD | 7 | 1 |
| Sex discordance | 0 | 0 |
| Duplicate/related samples | 32 | 6 |
|  |  |  |
| **SNP QC** |  |  |
| MAF < 0.01 | 37 | 1,866 |
| Call rate < 97% | 235 | 57 |
| HWE p-v < 1.0E-5 | 282 | 9,133 |
