# Supplementary material for: Ancestral origin of ApoE ε4 Alzheimer disease risk in Puerto Rican and African American populations
Source: PLoS Genet. 2018 Dec 5;14(12):e1007791. doi: 10.1371/journal.pgen.1007791 (PMC6281216; doi:10.1371/journal.pgen.1007791)
Supplement: S2 Table — (DOCX) [file pgen.1007791.s002.docx]

**S2 Table**. Counts of ε3 and ε4 alleles along African and European local ancestries in African-Americans and Puerto Ricans for cases and controls

|  | | | **Puerto Ricans** | | **African-Americans** | |
| --- | --- | --- | --- | --- | --- | --- |
| **Haplotype Ancestry** | **ApoE** | **Controls** | | **Cases** | **Controls** | **Cases** |
| **African** | **ε3** | **17** | | **21** | **2651** | **1273** |
|  | **ε4** | **13** | | **16** | **783** | **880** |
| **European** | **ε3** | **148** | | **111** | **695** | **301** |
|  | **ε4** | **11** | | **37** | **147** | **198** |
