# Supplementary material for: Ancestral origin of ApoE ε4 Alzheimer disease risk in Puerto Rican and African American populations
Source: PLoS Genet. 2018 Dec 5;14(12):e1007791. doi: 10.1371/journal.pgen.1007791 (PMC6281216; doi:10.1371/journal.pgen.1007791)
Supplement: S3 Table — (DOCX) [file pgen.1007791.s003.docx]

**S3 Table.** The complete list of the potential protective variants at the local ancestry blocks surrounding the ApoE gene

| **Marker ID** | **Base Position** | **Reference Allele** | **Alternative Allele** | **Bonferroni corrected**  **p-values** | |
| --- | --- | --- | --- | --- | --- |
|  |  |  |  | **CEU vs. YRI** | **JPT vs.YRI** |
| rs6857 | 45392254 | C | T | 8.44E-12 | 5.88E-06 |
| rs157585 | 45397512 | A | C | 1.31E-09 | 5.85E-07 |
| rs157588 | 45398264 | C | T | 1.31E-09 | 5.85E-07 |
| rs157590 | 45398716 | A | C | 1.31E-09 | 5.85E-07 |
| rs769449 | 45410002 | G | A | 1.31E-09 | 5.85E-07 |
| rs12721046 | 45421254 | G | A | 6.45E-09 | 3.71E-10 |
| rs111789331 | 45427125 | T | A | 6.45E-09 | 4.75E-09 |
| rs157584 | 45396899 | T | C | 6.45E-09 | 5.85E-07 |
| rs142042446 | 45386467 | G | GTAA | 3.02E-08 | 5.85E-07 |
| rs12972156 | 45387459 | C | G | 3.02E-08 | 5.85E-07 |
| rs12972970 | 45387596 | G | A | 3.02E-08 | 5.85E-07 |
| rs34342646 | 45388130 | G | A | 3.02E-08 | 5.85E-07 |
| rs71352238 | 45394336 | T | C | 3.02E-08 | 5.85E-07 |
| rs66626994 | 45428234 | G | A | 4.37E-08 | 4.97E-08 |
| rs846870 | 45086346 | C | T | 2.91E-07 | 0.017742653 |
| rs189902748 | 45171117 | C | A | 5.63E-07 | 0.007466462 |
| rs10417451 | 44644499 | T | C | 5.67E-07 | 0.024192929 |
| rs6509145 | 44644770 | T | G | 5.67E-07 | 0.024192929 |
| rs238418 | 45855262 | G | T | 1.18E-06 | 0.025359763 |
| rs1661198 | 45095973 | G | C | 1.42E-06 | 0.010478124 |
| rs1727777 | 45096299 | T | C | 1.42E-06 | 0.010478124 |
| rs12721051 | 45422160 | C | G | 3.62E-06 | 0.007466462 |
| rs2466421 | 44819582 | A | T | 7.47E-05 | 0.024767926 |
| rs2722718 | 44820462 | A | G | 7.47E-05 | 0.024767926 |
| rs2722719 | 44820527 | T | C | 7.47E-05 | 0.024767926 |
| rs2722735 | 44849565 | G | A | 7.47E-05 | 0.024767926 |
| rs2722736 | 44849714 | T | C | 7.47E-05 | 0.024767926 |
| rs2262860 | 44854752 | G | A | 7.47E-05 | 0.024767926 |
| rs1836273 | 44858873 | G | A | 7.47E-05 | 0.024767926 |
| rs2722677 | 44915902 | C | T | 0.000173518 | 0.024107011 |
| rs10409018 | 45029716 | C | T | 0.000250429 | 0.008898462 |
| rs1881042 | 45030209 | C | T | 0.000250429 | 0.008898462 |
| rs2075650 | 45395619 | A | G | 0.000461755 | 0.019296325 |
| rs34404554 | 45395909 | C | G | 0.000461755 | 0.019296325 |
| rs11556505 | 45396144 | C | T | 0.000461755 | 0.019296325 |
| rs71338767 | 45824148 | C | CTATTAT | 0.000530973 | 0.007466462 |
| rs12459496 | 45004042 | T | C | 0.001317958 | 0.000427694 |
| rs12977470 | 45004305 | G | C | 0.001317958 | 0.000427694 |
| rs2965164 | 45202052 | C | T | 0.001794968 | 0.002396553 |
| rs344816 | 45825626 | A | T | 0.001817523 | 0.000627501 |
| rs7260359 | 45814908 | C | T | 0.00595105 | 1.42E-05 |
| rs10402747 | 45815248 | T | C | 0.022298762 | 6.54E-05 |
| rs35930252 | 45816210 | T | TA | 0.022931569 | 0.000263173 |
