# Supplementary figures and images for: Ancestral origin of ApoE ε4 Alzheimer disease risk in Puerto Rican and African American populations
Source: PLoS Genet. 2018 Dec 5;14(12):e1007791. doi: 10.1371/journal.pgen.1007791 (PMC6281216; doi:10.1371/journal.pgen.1007791)

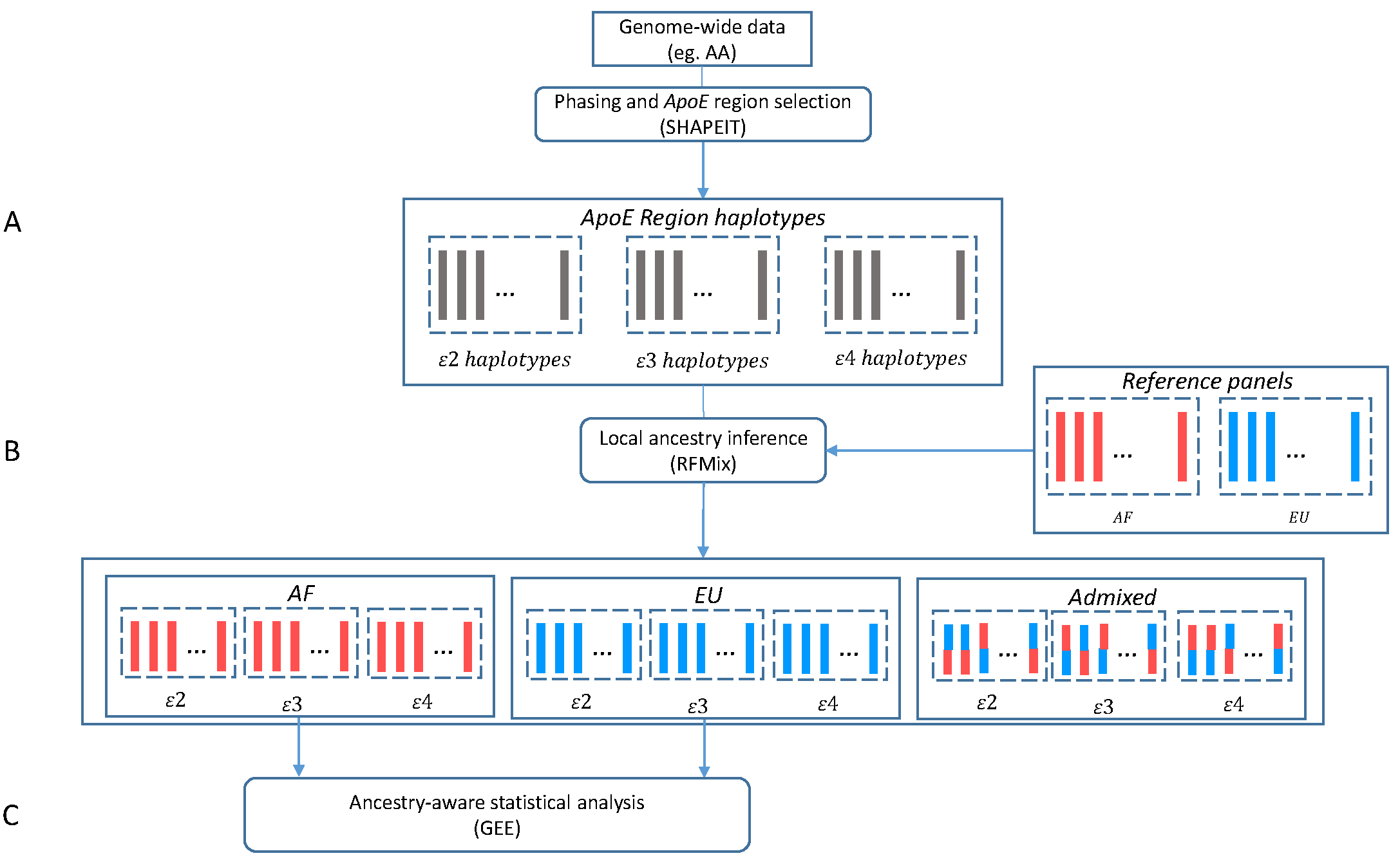

Supplement: S1 Fig — (A) Phasing, selecting the ApoE region, and classifying the haplotypes into three groups: ε2, ε3, ε4 haplotypes. (B) Building reference panels and inferring the local ancestry by using RFMix. Haplotypes classified as Reference ancestries ε2, ε3, ε4 haplotypes and admixed ε2, ε3, ε4 haplotypes. (C) Building statistical model to test the association of ancestry-aware ε4 alleles against ε3 within AF and EU subgroups, and admixed haplotypes within ApoE were excluded from analysis. (TIF) [file pgen.1007791.s004.tif]
